# Supplementary material for: Single-cell Hi-C data enhancement with deep residual and generative adversarial networks
Source: Bioinformatics. 2023 Jul 27;39(8):btad458. doi: 10.1093/bioinformatics/btad458 (PMC10403428; doi:10.1093/bioinformatics/btad458)
Supplement: btad458_Supplementary_Data [file btad458_supplementary_data.docx]

**Supplemental Data for**

***ScHiCEDRN*: Single-cell Hi-C data Enhancement with Deep Residual and Generative Adversarial Networks**

Yanli Wang^1,2^, Zhiye Guo^1,2^, and Jianlin Cheng^1,2*^

^1^Department of Electrical Engineering and Computer Science, University of Missouri, Columbia, MO 65211, USA; ^2^NextGen Precision Health Institute, University of Missouri, Columbia, MO 65211, USA.

*Corresponding author: chengji@missouri.edu

| **Table S1**. Comparison of the methods for enhancing the single-cell Hi-C data of drosophila cell 1 on *drosophia_cells_test_data* at different down-sampled ratios. | | | | | | | | | | | | |  |
| --- | --- | --- | --- | --- | --- | --- | --- | --- | --- | --- | --- | --- | --- |
|  | | **0.75 downsampled** | | | | **0.45 downsampled** | | | | | | |  |
| **Model** | | PSNR | SSIM | MSE | SNR | PSNR | SSIM | | MSE | | SNR | |  |
| Deephic | | 32.6319 | 0.9262 | 0.0006 | 1340.052 | 30.7695 | 0.9176 | | 0.0009 | | 1228.267 | |  |
| HiCSR | | **36.1308**** | **0.9554**** | **0.0003**** | **1875.1875**** | **32.615**** | **0.9526**** | | **0.0006**** | | **1555.2092**** | |  |
| HiCPlus | | 34.1131 | 0.9472 | 0.0005 | 1531.9993 | 26.33 | 0.9027 | | 0.0024 | | 1048.578 | |  |
| Loopenhance | | 30.7004 | 0.7893 | 0.001 | 989.2382 | 30.8793 | 0.8018 | | 0.001 | | 895.9495 | |  |
| **ScHiCEDRN** | | **37.1298*** | **0.9691*** | **0.0002*** | **2029.6637*** | **35.8235*** | **0.9578*** | | **0.0003*** | | **1743.115*** | |  |
|  | | **0.1 downsampled** | | | | **0.02 downsampled** | | | | | | |  |
| **Model** | | PSNR | SSIM | MSE | SNR | PSNR | SSIM | | MSE | | SNR | |  |
| Deephic | | 28.862 | 0.9097 | 0.0015 | 1122.2803 | 27.1336 | 0.9032 | | 0.0021 | | 1070.9573 | |  |
| HiCSR | | 30.173 | **0.9162**** | 0.0011 | **1211.6758**** | 28.0476 | **0.9042**** | | 0.0018 | | **1098.2803**** | |  |
| HiCPlus | | **31.9046**** | 0.9149 | **0.0007**** | 1202.5786 | **29.4927**** | 0.8968 | | **0.0013**** | | 990.645 | |  |
| Loopenhance | | 26.0136 | 0.5244 | 0.0025 | 664.7553 | 24.3224 | 0.4506 | | 0.0038 | | 506.221 | |  |
| **ScHiCEDRN** | | **32.6435*** | **0.9332*** | **0.0006*** | **1333.2994*** | **31.0117*** | **0.9189*** | | **0.0009*** | | **1198.6991*** | |  |
| Smaller the ratio, the sparser (noisier) the input data. The enhanced matrices are compared with the target matrices in terms of several image-based metrics. “*” and “**” denote the best and second-best results, respectively. | | | | | | | | | | | | |  |
| **Table S2**. Comparison of the methods for enhancing the single-cell Hi-C data of drosophila cell 2 on *drosophia_cells_test_data* at different down-sampled ratios. | | | | | | | | | | | | | |
|  | **0.75 downsampled** | | | | | **0.45 downsampled** | | | | | | | |
| **Model** | PSNR | | SSIM | MSE | SNR | PSNR | | SSIM | | MSE | | SNR | |
| Deephic | 29.3962 | | 0.8706 | 0.0011 | 908.9744 | 27.5517 | | 0.8546 | | 0.0017 | | 821.3755 | |
| HiCSR | **32.2567**** | | **0.9127**** | **0.0006**** | **1229.9049**** | **30.7281**** | | **0.9127**** | | **0.0008**** | | **1116.3223**** | |
| HiCPlus | 30.1586 | | 0.8984 | 0.0009 | 1011.9827 | 24.8154 | | 0.8385 | | 0.0032 | | 718.3521 | |
| Loopenhance | 26.9207 | | 0.7572 | 0.002 | 756.9747 | 27.0055 | | 0.7508 | | 0.002 | | 735.9614 | |
| **ScHiCEDRN** | **33.1761*** | | **0.942*** | **0.0005*** | **1369.3593*** | **31.6015*** | | **0.9198*** | | **0.0007*** | | **1160.4108*** | |
|  | **0.1 downsampled** | | | | | **0.02 downsampled** | | | | | | | |
| **Model** | PSNR | | SSIM | MSE | SNR | PSNR | | SSIM | | MSE | | SNR | |
| Deephic | 25.7155 | | 0.8395 | 0.0027 | 737.3024 | 25.15 | | **0.8371**** | | 0.0031 | | 721.76 | |
| HiCSR | 26.7969 | | 0.8496 | 0.0021 | 790.8455 | 25.5995 | | 0.8369 | | 0.0028 | | **736.1273**** | |
| HiCPlus | **27.8874**** | | **0.8553**** | **0.0016**** | **812.002**** | **26.5823**** | | 0.8333 | | **0.0024**** | | 702.1099 | |
| Loopenhance | 24.6288 | | 0.4671 | 0.0032 | 594.7316 | 23.3519 | | 0.4076 | | 0.0048 | | 471.8987 | |
| **ScHiCEDRN** | **28.4691*** | | **0.8699*** | **0.0014*** | **864.6344*** | **27.2431*** | | **0.8506*** | | **0.0019*** | | **784.5135*** | |
| Smaller the ratio, the sparser (noisier) the input data. The enhanced matrices are compared with the target matrices in terms of several image-based metrics. “*” and “**” denote the best and second-best results, respectively | | | | | | | | | | | | | |

| **Table S3**. Comparison of the methods for enhancing the population Hi-C data on two population Hi-C datasets. | | | | | | | | |
| --- | --- | --- | --- | --- | --- | --- | --- | --- |
|  | ***human_population_test_data*** | | | | ***drosophia_population_test_data*** | | | |
| **Model** | PSNR | SSIM | MSE | SNR | PSNR | SSIM | MSE | SNR |
| Deephic | **43.9108**** | 0.9764 | **0.00004**** | **300737.8**** | **46.9136**** | 0.9965 | **0.00002**** | **26316.4**** |
| HiCSR | 40.6342 | **0.9905**** | 0.00009 | 270261.1 | 44.0789 | 0.9948 | 0.00004 | 13237.0 |
| HiCPlus | 43.1320 | 0.9811 | 0.00005 | 288746.4 | 45.3919 | **0.9975**** | 0.00003 | 25221.5 |
| Loopenhance | 35.2915 | 0.7998 | 0.00027 | 74560.3 | 38.4085 | 0.9248 | 0.00013 | 3785.8 |
| **ScHiCEDRN** | **50.1347*** | **0.9983*** | **0.00001*** | **475441.0*** | **52.2230*** | **0.9994*** | **0.000005*** | **26543.8 *** |
|  | | | | | | | | |


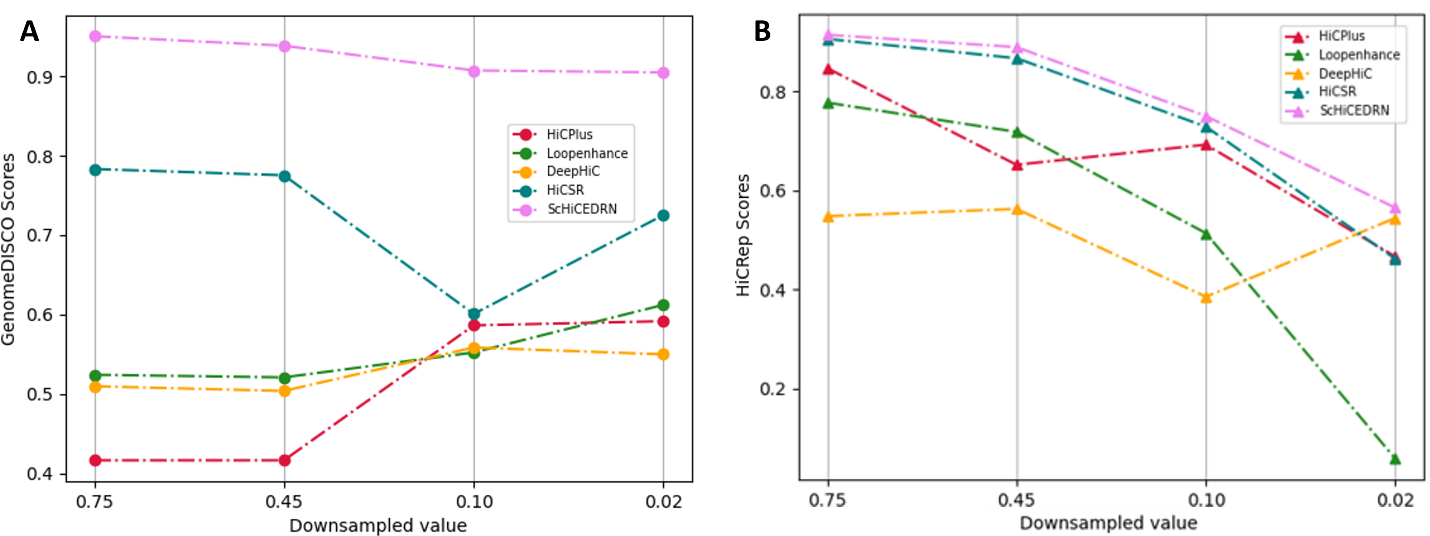


**Fig. S1**. The HiC reproducibility scores: (**A**) GenomeDISCO average scores and (**B**) HiCRep average scores of the five methods on single-cell Hi-C data of Drosophila cell 1 from *drosophila_cells_test_data* across different down-sampled ratios, respectively.


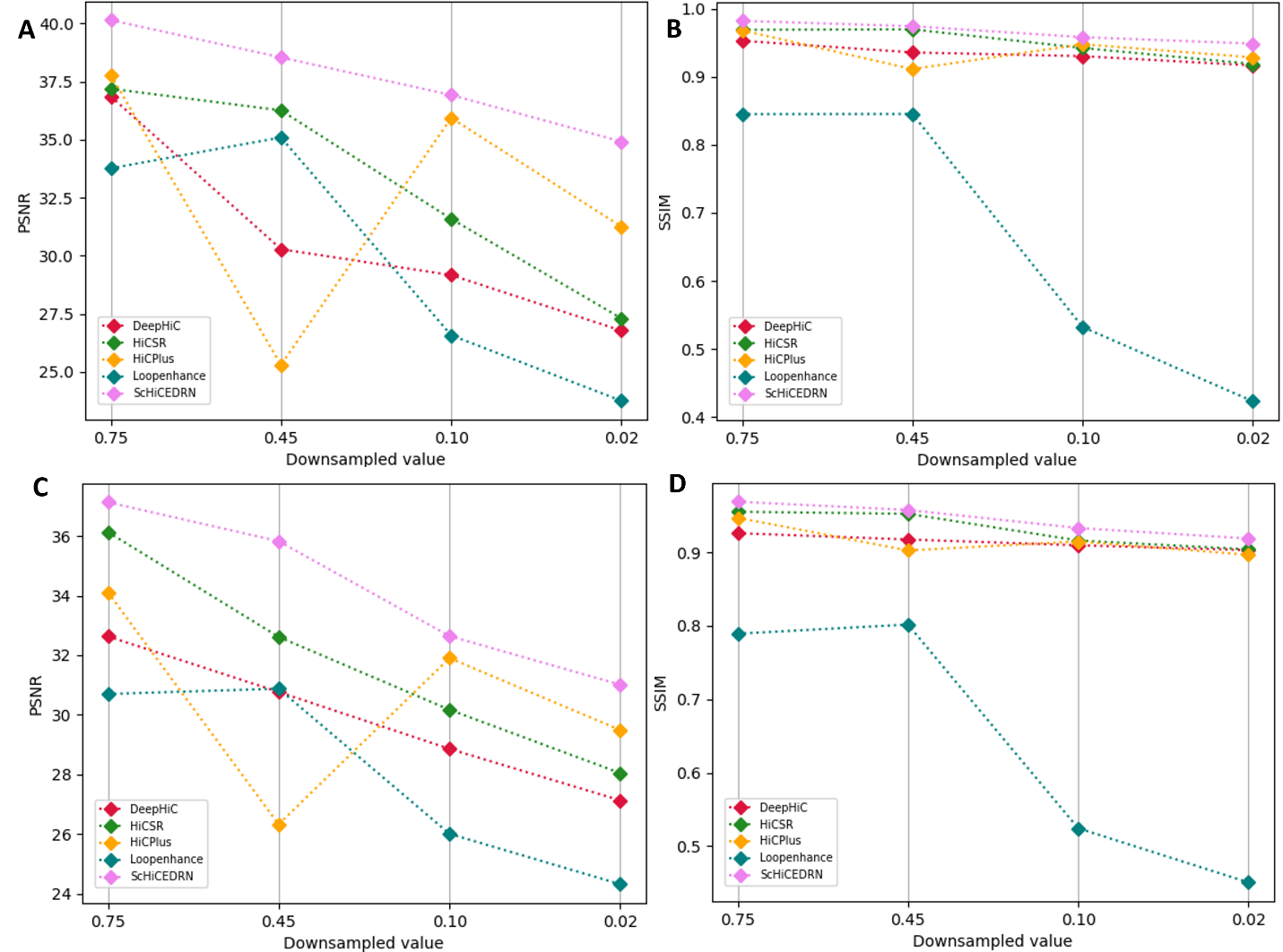


**Fig. S2.** The average PSNR and SSIM on enhancing the single-cell Hi-C data of human cell 3 from *human_cells_2_3_test_data* and Drosophila cell 2 from *drosophila_cells_test_data* at different down-sampled ratios, respectively. **(A)** PSNR for human cell 3, **(B)** SSIM for human cell 3, **(C)** PSNR for Drosophila cell 2, and **(D)** SSIM for Drosophila cell 2.


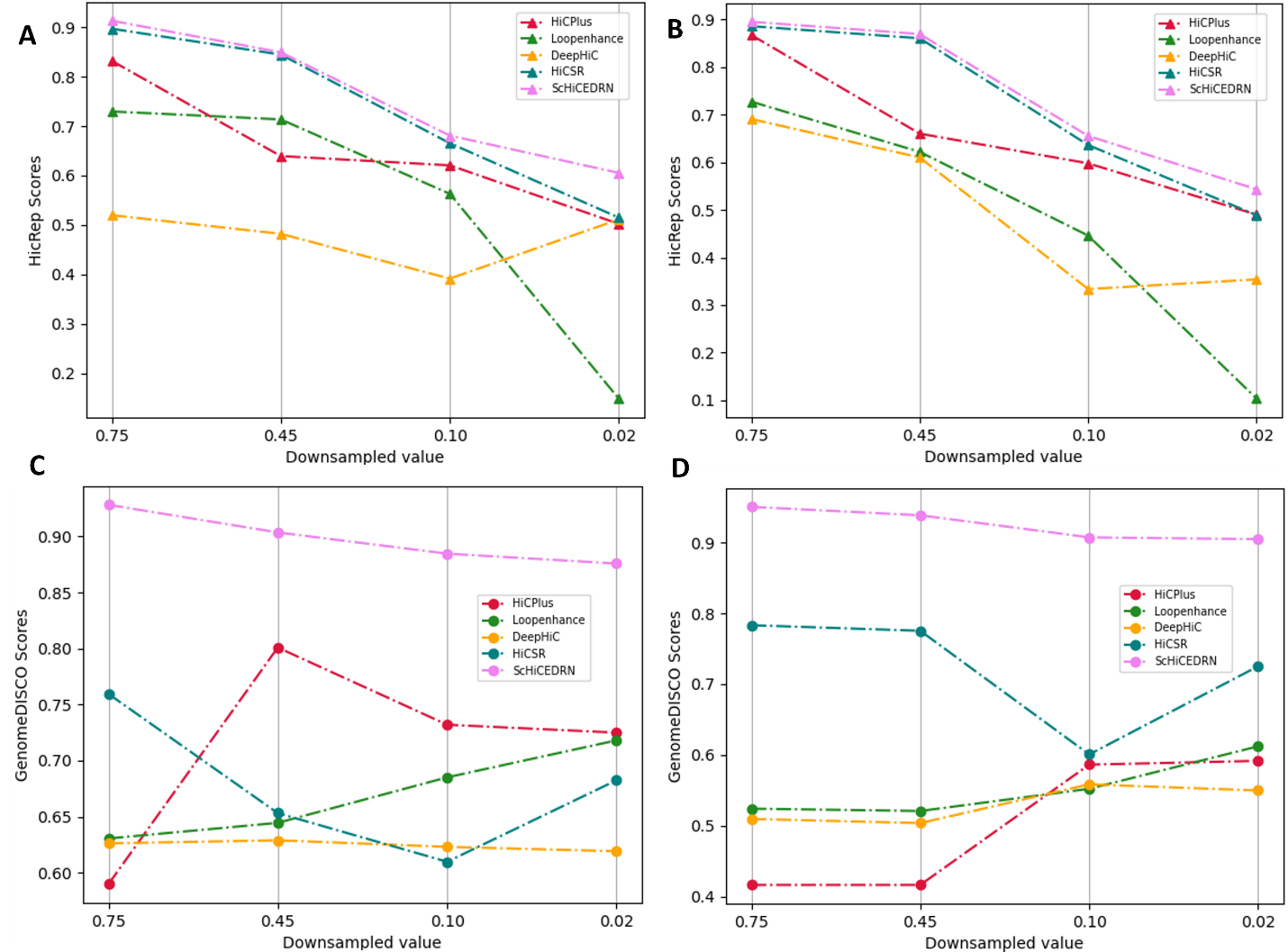


**Fig. S3**. The HiC reproducibility scores: HiCRep and GenomeDISCO average scores of the five methods on single-cell Hi-C data of human cell 3 from *human_cells_2_3_test_data* and Drosophila cell 2 from *drosophila_cells_test_data* across different down-sampled ratios, respectively. **(A)** HiCRep for Human cell 3, **(B)** HiCRep for Drosophila cell 2, **(C)** GenomeDISCO for Human cell 3, and **(D)** GenomeDISCO for Drosophila cell 2.


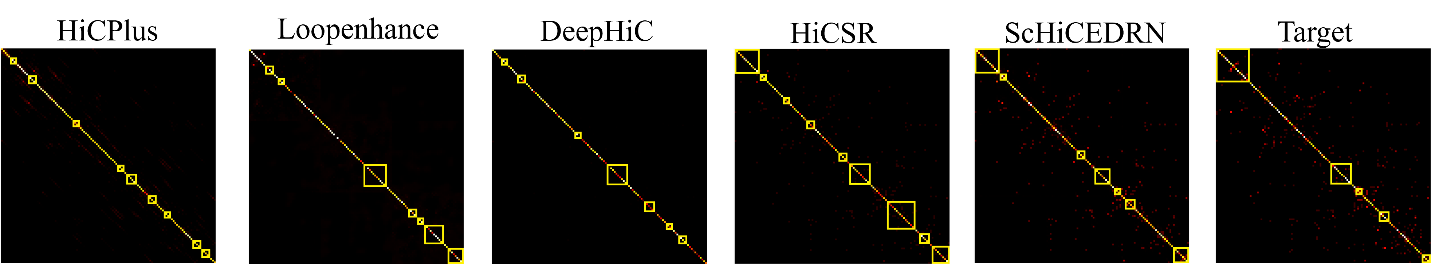


**Fig. S4**. The visualization of TADs extracted from the single-cell chromosomal contact matrices of the region 73.60-78.40 Mb of Chromosome 12 enhanced by the five methods and from the Target matrix (real high-resolution single-cell Hi-C chromosomal contact matrix). The TADs were identified and visualized by GenomeFlow. On each map, the bright yellow boxes along the diagonal represent predicted TADs. It is shown that the TADs identified from the matrix enhanced by ScHiCEDRN are more similar to the ones from Target than the other four methods.


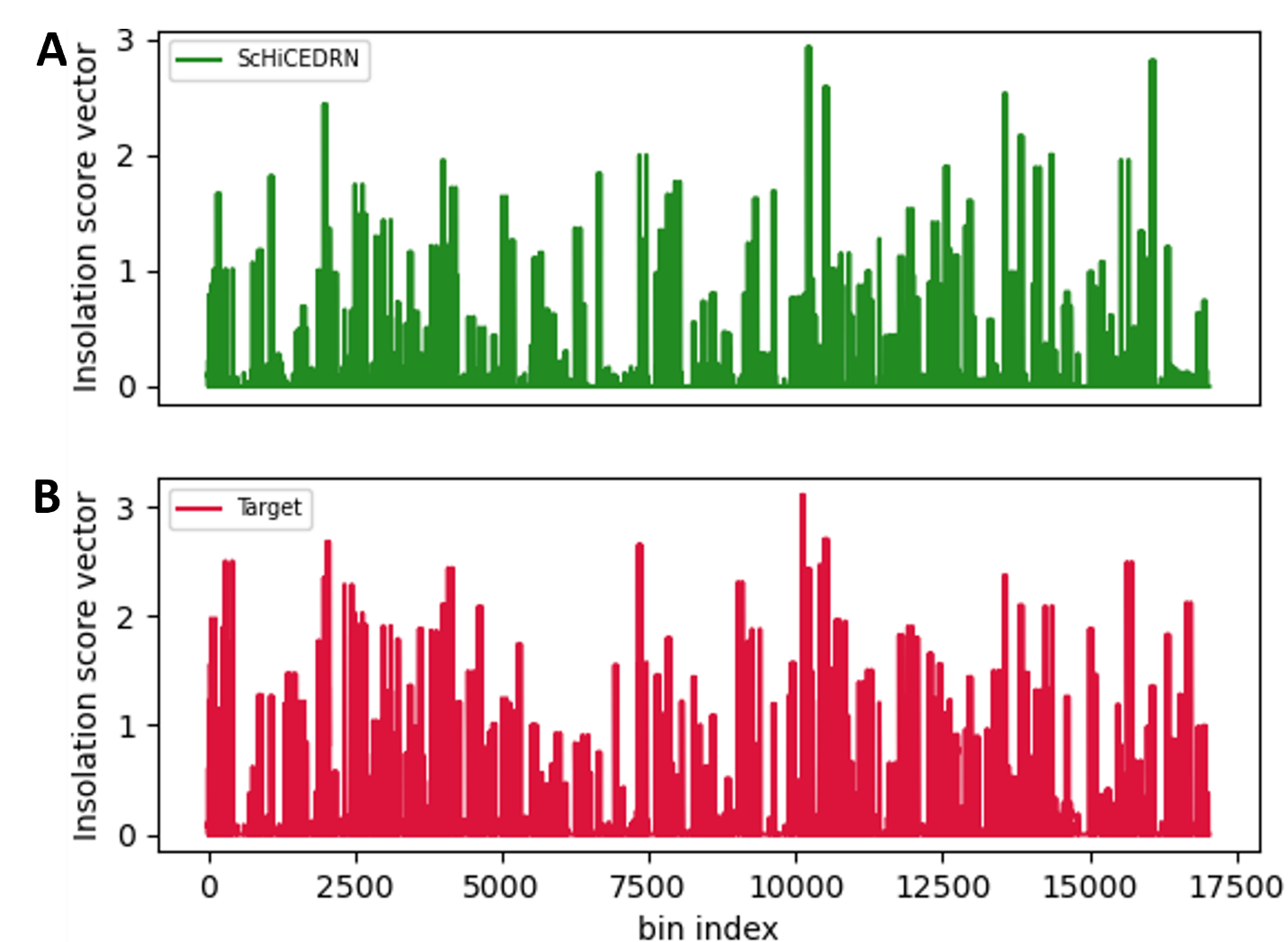


**Fig. S5**. The plots of the insulation score vectors along the chromosomal bins. (**A**) The plot of the aggregated insulation score vector computed from the single-cell Hi-C chromosomal contact matrices enhanced by ScHiCEDRN for the human cell 2 from *human_cells_2_3_test_data*; (**B**) the plot of the insulation score vector computed from the Target (the real high-resolution single-cell Hi-C chromosomal contact matrix) for the human cell 2 from *human_cells_2_3_test_data*. For the simplicity, the insulation score vectors for Chromosomes 2, 6, 10, and 12 are plotted in the same plot by linking their chromosomal bins together sequentially. The two plots have similar insulation score patterns.


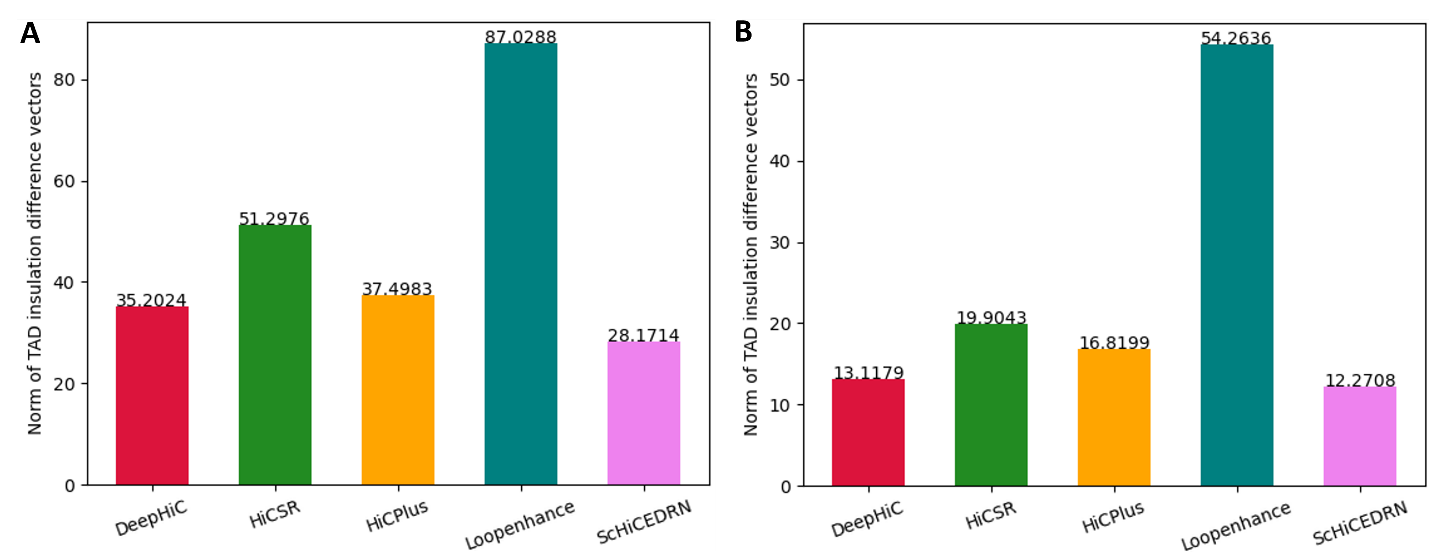


**Fig. S6.** The L2 norm difference between the TAD insulation vectors computed from the matrices enhanced by the five methods and the insulation vectors computed from the original high-resolution matrices on the two population Hi-C datasets. (**A**) On *human_population_test_data*, (**B**) On *drosophia_population_test_data*.


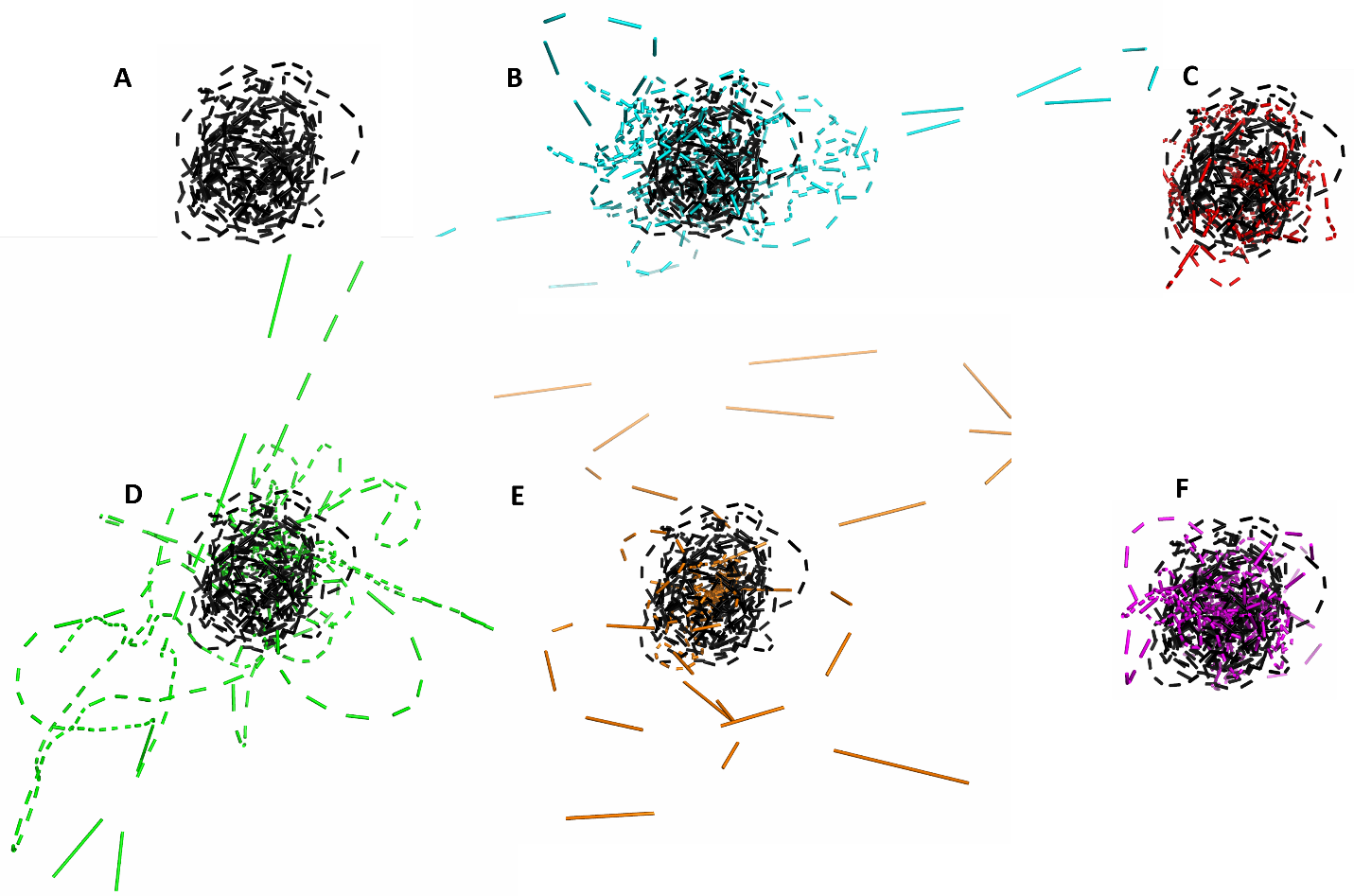


**Fig. S7**. The 3D chromosome conformations reconstructed from the single-cell Hi-C data enhanced with different methods against the conformation reconstructed from real single-cell Hi-C data for the same region 73.60-78.40 Mb of Chromosome 12. (**A**) The 3D conformation reconstructed from the real single-cell Hi-C data. (**B**) The 3D conformation with HiCPlus aligned against A. (**C**) The 3D conformation with DeepHiC aligned against A. (**D**) The 3D conformation with HiCSR aligned against A. (**E**) The 3D conformation with Loopenhance aligned against A. **(F)** The 3D conformation with ScHiCEDRN aligned against A.
